# Deuterated Nanopolymers for Renal and Lymphatic Imaging via Quantitative Deuterium MRI

**DOI:** 10.1021/acs.nanolett.4c03036

**Published:** 2025-01-22

**Authors:** Lisa M. Fries, Elton T. Montrazi, Hyla Allouche-Arnon, Felipe Opazo, Amnon Bar-Shir, Lucio Frydman, Stefan Glöggler

**Affiliations:** † NMR Signal Enhancement Group, Max Planck Institute for Multidisciplinary Sciences, 37077 Göttingen, Germany; ‡ Center for Biostructural Imaging of Neurodegeneration of the University Medical Center, 37075 Göttingen, Germany; § Department of Chemical and Biological Physics, Weizmann Institute of Science, Rehovot 76100, Israel; ∥ Department of Molecular Chemistry and Materials Science, Weizmann Institute of Science, Rehovot 76100, Israel; ⊥ Institute for Neuro- and Sensory Physiology, University Medical Center, 37075 Göttingen, Germany

**Keywords:** magnetic resonance imaging, deuterium imaging, dendrimers, *in vivo*, inflammation, molecular imaging

## Abstract

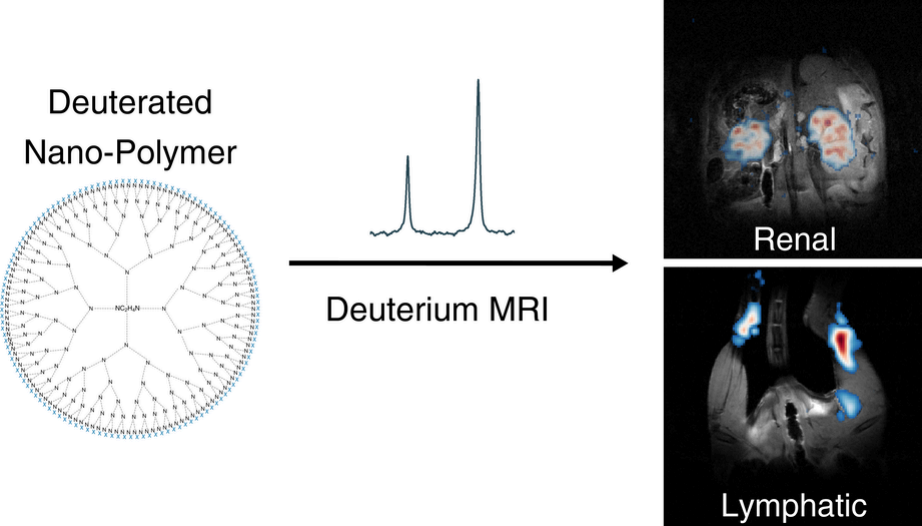

Deuterium (^2^H) MRI is an emerging tool for noninvasive imaging. We explore the integration of ^2^H MRI with deuterated multifunctional nanopolymers for deuterated particle imaging (DPI). To this end, amine-terminated G5-polyamidoamine (PAMAM) dendrimers were labeled with deuterated acetyl surface groups, leading to highly ^2^H-loaded bioparticles, making them ideal for imaging studies. The accumulation of ∼5 nm PAMAM dendrimers in the kidneys could then be seen by ^2^H MRI with high submillimeter resolution. The natural abundance HDO signal provided an internal concentration reference to these measurements, leading to quantitative dynamic maps showing distinct nanopolymer uptakes within the renal compartments. Further, these nanopolymers allowed us to obtain *in vivo* maps of activity in the lymph nodes in an inflammatory rodent leg model, demonstrating these deuterated nanopolymers’ potential as a novel class of contrast agents for the quantitative mapping of physiological processes.

Deuterium MRI is an emerging method for noninvasive imaging, most frequently undertaken with deuterated [6,6′-^2^H_2_]glucose to quantify and image cellular metabolism *in vivo*.^1−3^ By contrast to ^1^H-MRI, ^2^H-MRI agents provide a platform for imaging relatively few signals that have the unique potential to lead to metabolic hotspots. In comparison to most noninvasive imaging modalities, the signal of the deuterated contrast agent can be referenced to the natural abundance HDO signal present *in vivo* to provide a quantitative description of concentrations and fluxes.^2^ Although there are no endogenous molecules, nanopolymer-based imaging agents could offer extensive adaptability to ^2^H MRI, making them well suited as customized reporting agents. This study explores the use of deuterated nanopolymers—more specifically, heavily deuterated dendrimers—as potential contrast and reporting deuterium MRI agents for deuterium particle imaging (DPI).

Dendrimers are a major class of polymers encompassing a central core and side chains branching out radially from this core that can be adjustable in size.^4−7^ The ability to modify and optimize the surface groups of the branches expands the manipulation of dendrimers, thus customizing them for the desired imaging application.^8,9^ Poly(amidoamine) (PAMAM) dendrimers are the most commonly used agents, as they are highly soluble in water and can serve as nanodrug carriers and their primary amine surface groups present suitable attachment sites for other molecules.^10^ Despite their widespread application, dendrimers present significant toxicity concerns.^7,11^ Modifying surface groups is essential to mitigating toxicity and improving biocompatibility. Attaching neutral surface groups to PAMAM dendrimers effectively reduces cytotoxicity by reducing interactions between positively charged dendrimers and negatively charged biological membranes in vivo.^12^ The use of PAMAM dendrimers for diagnostic imaging was demonstrated nearly a decade ago by introducing Gd ligands as surface groups to enhance the dendrimer’s contrast.^13^ These PAMAMs showed a prolonged circulating time in the body, making them suitable contrast agents for imaging slower dynamic processes and biodistributions.^14−20^ Even though these dendrimers were highly sensitive, they generated nonspecific, strong background signals and could not be quantified *in vivo*. PAMAMs, wherein the metal-based ligands were substituted with radical ligands for the imaging of glioblastoma,^21^ as well as dendrimers oriented to chemical exchange saturation transfer (CEST) MRI^22,23^ and heteronuclear MRI^24−27^ have also been presented; still, these also present challenges to achieving accurate concentration characterizations.

To alleviate this handicap, we explored leveraging deuterium MRI, an alternative that has already been translated to human studies,^1,28−31^ for the quantitative detection of these dendrimers. To this end, generation 5 PAMAM dendrimers were modified with deuterated acetyl groups at their surfaces, leading to highly sensitive ^2^H MRI agents with 326 equiv of deuterons per dendrimer, all in rapidly rotating, internally flexible methyl groups. The attachment of acetyl groups reduces cytotoxicity and enhances biocompatibility, enabling the use of higher-generation PAMAM dendrimers, which would otherwise be more toxic without modification.^12^

A generation 5 dendrimer was chosen to be able to perform renal and lymphatic imaging with the same nanopolymer and to ensure a higher concentration of deuterium, even in cases of lower accumulation.^6,9^ The biodistribution of these deuterated nanopolymers was then monitored with customized high-sensitivity steady-state free precession (SSFP) methods customized to ultrahigh field (15.2 T) MRI. This enabled us to perform submillimeter (in-plane) *in vivo* imaging at submicromolar particle concentrations. Distinct distributions and dynamics in the different anatomical substructures of the kidneys were then clearly seen by ^2^H MRI and quantified using the natural abundance HDO signature. The deuterated nanopolymer was also shown to serve as MRI tracers, enabling the noninvasive mapping of inflammatory activity, as demonstrated with a model of leg inflammation induced by *Mycobacterium tuberculosis* for which ^2^H MRI showed clear lymphatic node swelling and uptake. Further potential uses of this approach for biomedical applications are discussed.

The nanopolymers were synthesized by modifying the surface of a G5-PAMAM dendrimer with a deuterated acetyl group (SI).^32^ A schematic 2D structure of the final nanopolymer (∼34 kDa) is shown in Figure 1a. The particle size was measured using dynamic light scattering (DLS) and transmission electron microscopy (TEM), yielding a value of 4.7 ± 0.2 nm (Figure S1). The observed reduction in size relative to G5-PAMAM is consistent with the existing literature, attributed to diminished electrostatic interactions resulting from acetylation.^33,34^ Due to the neutralization of surface groups, the zeta potential is also reduced to a zeta potential of 22 ± 2 mV compared to the unmodified version of PAMAM dendrimers (43.3 ± 0.7 mV^35^).

**Figure 1:**
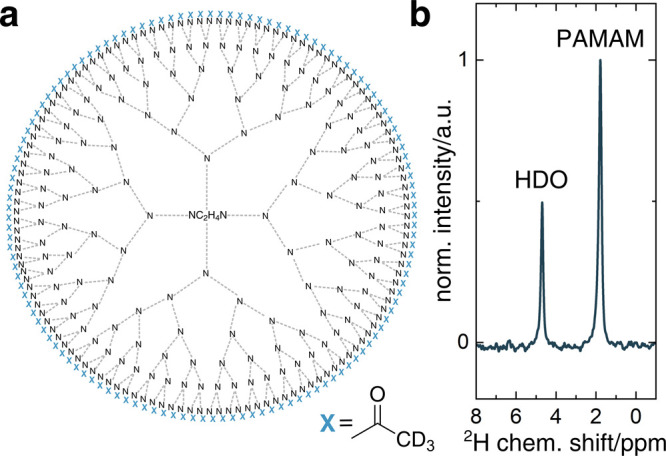
Schematic molecular structure and NMR spectrum of PAMAM-G5-Ac-d_3_. (a) 2D schematic structure of a PAMAM-G5 dendrimer modified with acetyl-d_3_ surface groups (PAMAM-G5-Ac-d_3_). (b) ^2^H NMR spectrum of 130 nM PAMAM-G5-Ac-d_3_ (1.9 ppm) in H_2_O (4.8 ppm).

The ^2^H NMR spectrum of PAMAM-G5-Ac-d_3_ dissolved in water produced one distinct peak at 1.9 ppm, which was spectrally well separated from the water peak at 4.8 ppm (Figure 1b). All of the deuterated groups in the molecule display a single peak with a 1.7 Hz line width. By comparing the integral of PAMAM-G5-Ac-d_3_ to the HDO integral with a natural abundance of 16.6 mM, the surface acetylation could be determined to be 85% (Figure S2). A phantom containing 0.13 mM PAMAM-G5-Ac-d_3_ in 2% (w/v) aqueous agarose was prepared for the measurement of T_1_ and T_2_ times mimicking relaxation in biological tissue. The relaxation times of PAMAM-G5-Ac-d_3_ and water are summarized in Table 1 (Figure S3). For the deuterium MRI studies, a CSI-SSFP sequence was used as shown in Figure 2a.^36^ Since the SSFP signal is dependent on the T_1_/T_2_ ratio and is maximized when this ratio is ∼1, the relaxation properties of PAMAM-G5-Ac-d_3_ are nearly ideal for studies using this sequence (details in Figure S4). Figure 2 illustrates this with a CSI-SSFP study on a two-component phantom, involving one tube made of a 16.5 mM D_2_O dissolved in H_2_O (H_2_O contains a concentration of 17 mM deuterium at natural abundance) and 2% (w/v) agarose and the other containing 0.13 mM PAMAM-G5-Ac-d_3_ dissolved in 2 wt % agarose in aqueous solution. Both tubes ended up containing 50 mM deuterium concentrations of HDO and PAMAM, respectively. Although the deuterium concentrations in both phantoms are equal, the intensity of the HDO image is lower compared to that of the PAMAM image. This is because T_1_ > T_2_ for water, resulting in a suboptimal signal. SSFP data acquired at 15.2 T MRI using the scheme in Figure 2a and processed as described in SI can then generate two clearly resolved ^2^H-MR images: one for HDO at δ = 4.8 ppm and one for PAMAM at δ = 1.9 ppm. The spectral separation between HDO and PAMAM (2.9 ppm) and the high fields employed yield two clear ^2^H-MR images devoid of cross-talk among the resonances (Figure 2c,d).

**Table 1:** Relaxation Times of PAMAM-G5-Ac-d_3_ and HDO at 14.1 T (600 MHz ^1^H)

**Compound**	**T**_**1**_	**T**_**2**_	**Shifts**
PAMAM-G5-Ac-d_3_	0.135 s	0.120 s	1.9 ppm
Water	0.546 s	0.060 s	4.8 ppm

**Figure 2:**
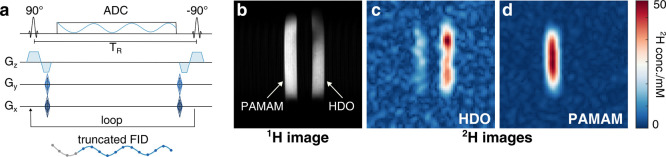
CSI-SSFP for the deuterium imaging of phantoms containing HDO and PAMAM-G5-Ac-d_3_. (a) Slice-selective CSI-SSFP sequence used in this study. (b) ^1^H FLASH image of the phantom made of two 5 mm NMR tubes containing a 50 mM ^2^H concentration of PAMAM-G5-Ac-d_3_ with 17 mM natural abundance HDO (360 nM in PAMAM concentration; left tube) and 50 mM HDO in 2 wt % agarose (right tube) with a FOV of 42 × 42 mm^2^. (c) HDO image and (d) PAMAM-G5-Ac-d_3_ image with an FOV of 42 × 42 mm^2^, resolution of 1.3 mm, and acquisition time of 6 min. See the SI for further experimental details.

Since our PAMAM-G5-Ac-d_3_ particles are ∼5 nm in size, they are likely to be cleared through the kidneys. This makes them suitable agents for monitoring renal activity *in vivo*.^15^ Prior to the *in vivo* experiments, stability tests were performed to prove that PAMAM-G5-Ac-d_3_ particles are stable in culture medium for at least 6 days, eliminating the risk of decay product-related side effects (Figure S5). For the *in vivo* experiments, eight-week-old female SJL/J mice (*n* = 3) were injected intravenously with 300 nmol of PAMAM-G5-Ac-d_3_ dissolved in 200 μL of PBS. ^2^H-MR images centered on the kidneys were acquired every minute after injection to follow the real-time kinetics of renal uptake with a matrix size of 16 × 16 (Figure 3a and Figure S3). All images were corrected for the ^2^H peak arising at ca. 1.3 ppm from abdominal fat by subtracting from the postinjection data a preinjection image (details in SI). Figure 3b shows the overlay of the resulting ^2^H-MRI PAMAM-G5-Ac-d_3_ image acquired 15 min after injection on the anatomical ^1^H MR image, clearly showing that PAMAM-G5-Ac-d_3_ accumulates in the kidneys. Using the intrinsic HDO signal of the bladder (^2^H concentration of 16.6 mM)^37^ as a reference, the amount of nanopolymers accumulated in the kidneys was estimated, leading to the results shown in Figure 3c for all three mice. (For details on quantification, see the SI.) A variance in dendrimer uptake between the left and right kidneys was observed in all three mice. After renal clearance, the nanopolymers can be found in the bladder (Figure S8).

**Figure 3:**
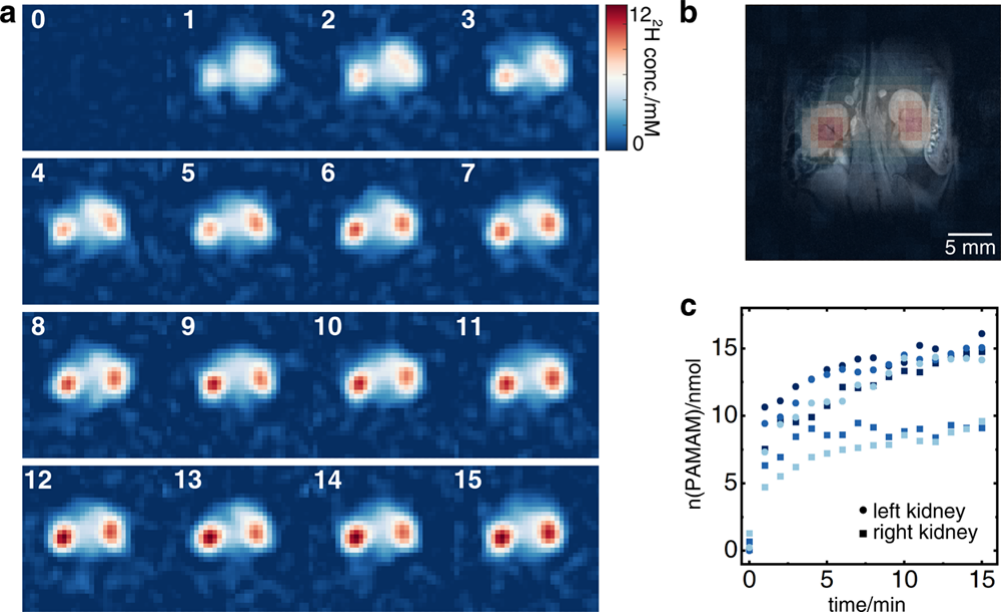
Renal uptake of PAMAM-G5-Ac-d_3_*in vivo* in mice (*n* = 3). (a) ^2^H images of PAMAM-G5-Ac-d_3_ for real-time imaging of renal uptake after an intravenous injection of 300 nmol. CSI-SSFP images (16 × 16 matrix) were acquired every minute with a resolution of 2.6 mm and an FOV of 42 × 42 mm^2^. (b) ^1^H/^2^H overlay of the deuterium MRI image of PAMAM-G5-Ac-d_3_ recorded 15 min after injection and the anatomical ^1^H RARE image. (c) Kinetic data showing PAMAM-G5-Ac-d_3_ accumulation within the first 15 min in the animal kidneys of all three mice correlated to the intrinsic HDO peak.

Besides capturing the dynamic processes of the rapid renal uptake, the high label loading and favorable relaxation properties of the PAMAM-G5-Ac-d_3_ nanopolymers enabled us to significantly enhance the resolution of the deuterium images. This in turn made it possible to map the distribution of the deuterated nanopolymers within the kidneys in further detail. Figure 4a and Figure S6 present high-resolution deuterium MRI images collected with 0.65 mm in-plane resolution and acquired 0.5, 6, and 24 h after the nanopolymer injection. The overlay of the ^1^H anatomical RARE image and the ^2^H PAMAM-G5-Ac-d_3_ image at 0.5 h evidence an accumulation of the nanopolymers within the renal pelvis, while after 6 h the dendrimers have spread throughout the kidneys. A substructure is still slightly visible, with a higher concentration in the medulla compartments. After 24 h, the deuterated dendrimers are even more diffuse throughout the kidney. Figure 4b shows the three images taken after 0.5, 6, and 24 h referenced to the highest intensity of the three images, visualizing the clearance of the nanopolymers from the kidneys to the bladder. A quantitative depiction of the global renal clearances for the three mice is shown in Figure 4c, evidencing a median clearance rate of (6 ± 3) nmol/h. Notice that after 24 h approximately 10% of the total injection concentration (300 nM) can still be detected in the kidneys, indicating a prolonged stay time within the organism with a half-life of 11 h.

**Figure 4:**
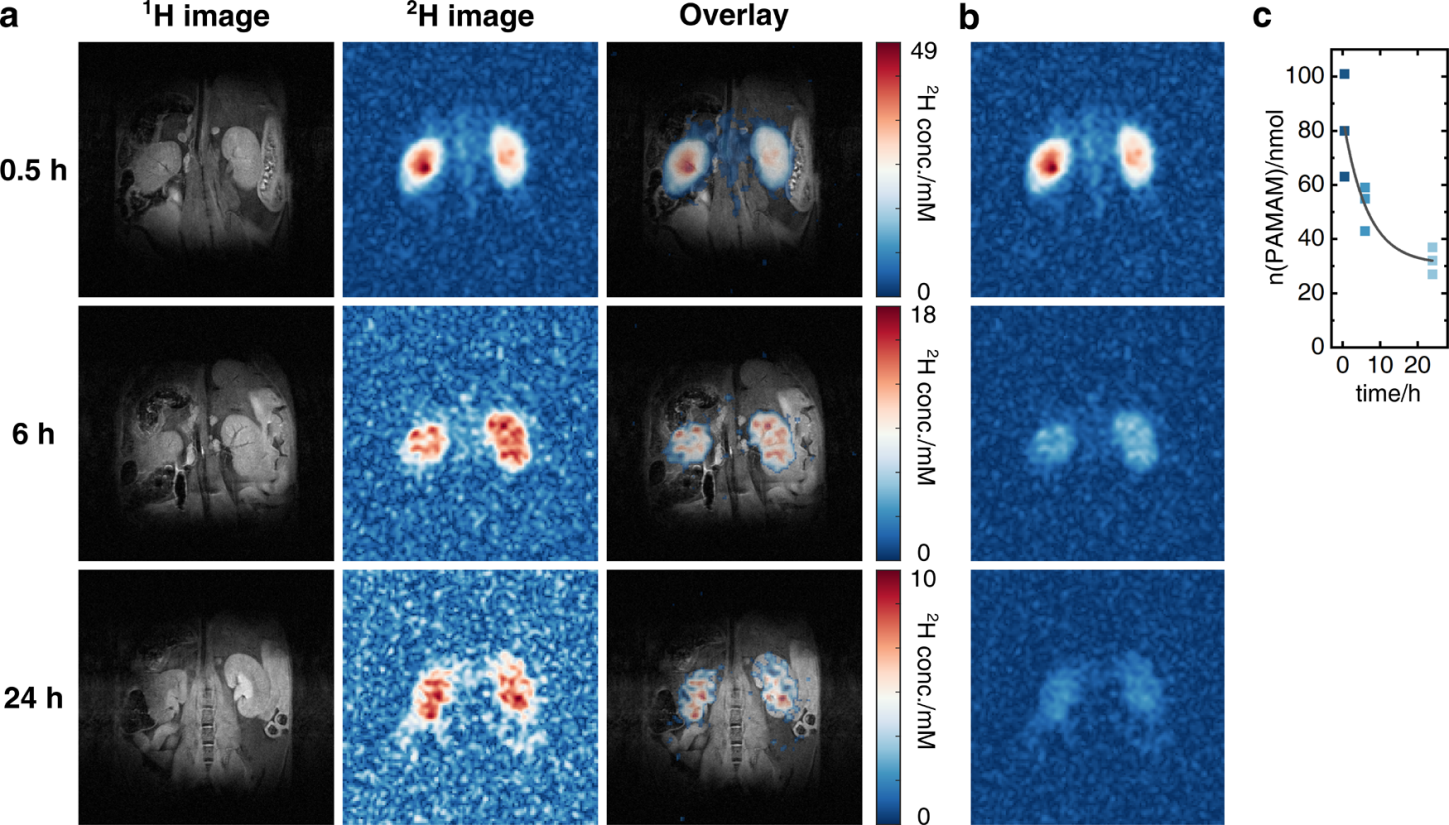
*In vivo* high-resolution deuterium MRI of PAMAM’s uptake and clearance in mice kidneys. (a) ^1^H anatomical RARE image and respective ^2^H images of PAMAM-G5-Ac-d_3_ and the ^1^H/^2^H overlay 0.5, 6, and 24 h after intravenous injection. All images were referenced to their maximum intensity. CSI-SSFP images (64 × 64 matrix) were acquired with a resolution of 0.65 mm, an FOV of 42 × 42 mm^2^, and an acquisition time of 24 min. (b) Visualization of PAMAM-G5-Ac-d_3_’s renal clearance by referencing the intensities of all images to the highest signal. (c) Amounts of PAMAM-G5-Ac-d_3_ within the kidneys of all three mice after 0.5, 6, and 24 h, with an exponential fit of slope (6 ± 3) nmol/h.

As nanopolymers are known to interact with the immune system,^38,39^ we set out to assess whether PAMAM-G5-Ac-d_3_ could also serve as a minimally invasive tracer for mapping inflammatory activity via ^2^H MRI. To this end, the footpads of eight-week-old female SJL/J mice (*n* = 3; see the SI for further information) were subcutaneously injected with 50 μL of an immunogenic emulsion, inducing a local inflammation in the right hind. Seven days after the injections, when extensive inflammatory activity was observed in the lymph nodes in the proximity of the immunized hind (Figure 5a and Figure S9), 20 μL of PAMAM-G5-Ac-d_3_ dissolved in PBS (100 mg/mL) was injected subcutaneously in the right and left footpads. One hour after injection, ^2^H MRI was performed to observe the accumulation of dendrimers in the lymphatic system. The ^1^H/^2^H overlay of the HDO image (Figure 5b) shows a distinct spot solely at the injection site of the untreated leg. Figure 5c confirms that the injected solution of deuterated dendrimers was not distributed in the lymphatic system or tissue of the untreated leg, whereas in the inflamed leg a specific accumulation in the two major lymph nodes of the leg (1: popliteal and 2: subiliac, as marked in Figure 5a) is clearly visible. The accumulation of nanopolymers is higher in the popliteal lymph node due to its proximity to the injection site. This could be attributed to macrophage-mediated internalization of the injected nanopolymer (Figure S10).

**Figure 5:**
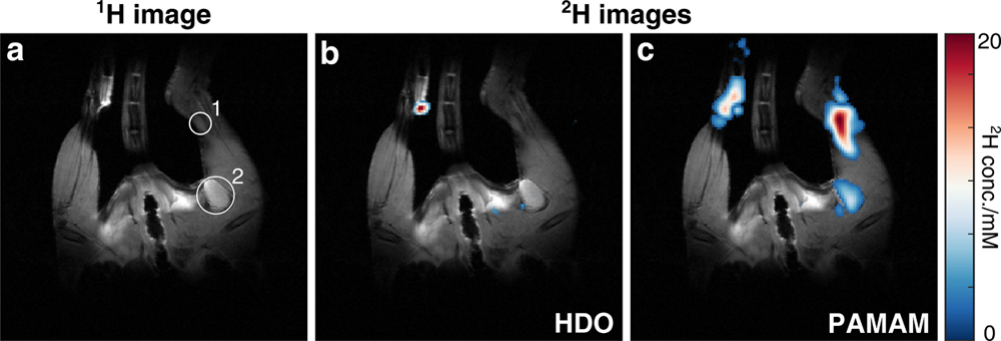
*In vivo*^2^H MRI study of immunized mice. Seven days postimmunization, each leg of the mice (*n* = 3) was subcutaneously injected with 100 nM PAMAM-G5-Ac-d_3_. (a) ^1^H anatomical image. The lymph nodes (1: popliteal; 2: subiliac) of the inflamed leg are marked with white circles. (b) ^1^H/^2^H overlay of the HDO image recorded with CSI-SSFP, showing a signal solely at the injection site of the untreated leg. (c) ^1^H/^2^H overlay of the deuterated dendrimer image, showing signals at the injection sites as well as at the lymph nodes. ^2^H images in (b) and (c) were acquired with a resolution of 0.65 mm and an FOV of 42 × 42 mm^2^.

To provide further evidence for macrophage internalization, we conducted experiments using confocal microscopy on the J774A.1 macrophage cell line, and it reveals the presence of fluorescently labeled nanopolymers within the macrophages (Figure S11). To confirm that this is physiological endocytosis, we observed colocalization when fluorescently labeled PAMAM-G5-Ac-d_3_ was incubated with fluorescently labeled dextran, an established marker for endocytosis. While the provided experiments are indicative of the proposed mechanism of uptake, further *in vivo* studies need to be undertaken to prove the biological concept. We are planning on pursuing this in future studies, as the combined use of fluorescent and deuterated dendrimers could not only serve to understand biological mechanisms but may also be a new opportunity to design bimodal contrast agents combining optical imaging and MRI.

The research on deuterated nanopolymers for *in vivo* DPI of renal and inflammatory conditions has produced encouraging outcomes, showcasing their high sensitivity and quantification capabilities. However, certain limitations need to be addressed before progressing to clinical translation. The quantification abilities are limited by the use of a surface coil, which affects the precision of flip angle measurements. In addition, quantification relies on the assumed T_1_ and T_2_ values for HDO, which may vary *in vivo* across different organs and tissues. This variability could introduce inaccuracies under physiological conditions. Future studies will address these limitations by utilizing a volume coil for more precise flip angle measurements and measuring HDO properties in the bladder in vivo prior to ^2^H administration to improve the quantification accuracy.

Our study was conducted at a high magnetic field strength of 15.2 T, which leverages the sensitivity of such fields and the brief acquisition times required to differentiate resonances in ^2^H MRSI. However, these field strengths are not clinically available. Ongoing research is exploring the use of parallel receiving structures and alternative image processing pipelines to mitigate the limitations associated with lower field strengths. Additionally, employing structures with higher deuterium loads could offset the SNR and enhance the deuterium intensity in specific compartments. Translational research is currently addressing these challenges, aiming to make our technique clinically viable at lower field strengths.

Despite their potential, a detailed understanding of dendrimer interactions with biological systems remains incomplete. Key aspects such as absorption, distribution, metabolism, and excretion require further investigation to fully assess their safety and efficacy.^40,41^ Comprehensive studies addressing these parameters are essential to advancing their use in clinical applications.

A future direct scientific application could involve using these deuterated nanopolymers for renal disease imaging, providing insights into variations in uptake or distribution within the kidneys.^42,43^ This will also require further investigation of renal uptake in wild-type mice, as variability in renal uptake was observed among the three mice measured. The variability between left and right kidney uptake has also been observed in PET studies.^44,45^

Further, the surface could be modified with targeting moieties designed for specific malignancies, including those pertinent to cancer immunotherapy.^38,46^ This approach is in line with the increasing focus on improving the specificity and effectiveness of therapeutic agents. Additionally, the application of deuterated nanopolymers in addressing the challenges associated with cell tracking and the blood–brain barrier (BBB) is of significant interest. The modification of dendrimers to enhance their BBB permeability offers a substantial opportunity, especially for the imaging and treatment of neurodegenerative diseases.^47,48^

The broad applicability of nanopolymers in biomedicine necessitates an extensive evaluation of various factors such as nanopolymer type, particle size, and surface group modification, among others. These considerations are crucial for determining the most suitable nanopolymers for nanomedical applications. Furthermore, the dual functionality of deuterated nanopolymers as both nanocarriers and MRI imaging agents represents a notable advantage. This dual capability facilitates the monitoring of drug pathways *in vivo*, yielding important information about drug distribution and efficacy. The integration of nanotechnology with medical imaging through the development of deuterated nanopolymers and DPI offers substantial promise for enhancing diagnostic and therapeutic methods in healthcare.

This study introduced deuterated particle imaging (DPI) by using deuterated nanopolymers for tracing renal uptake and clearance and as inflammation markers using *in vivo*^2^H spectroscopic MRI. The deuterated PAMAM dendrimer (PAMAM-G5-Ac-d_3_) was chosen for this for its versatility, high mass sensitive, and easy quantification by SSFP MRI. The results successfully demonstrate these agents’ usefulness in real-time renal uptake monitoring and high-resolution imaging of kidney substructures as well as a capability to identify inflammation sites postsystemic administration, illustrated in particular through hotspot MRI mapping of lymphatic uptake. While complete clearance of these nanopolymers remains a challenge for human application, no evidence of acute toxicity was observed within the time scale of our experiments. This work advances molecular imaging by providing a platform for quantitative noninvasive diagnostics with potential extensions to theranostic applications. Future studies should focus on long-term toxicity assessment and optimization of nanopolymer size to improve clearance and safety. With tailored surface modifications, these agents could be used for nanodrug delivery and other biomedical applications.

## Supporting Information Available

Details of the synthesis of the PAMAM; NMR and MRI sequences and image processing parameters employed; *in vivo* procedures; ancillary bioanalytical measurements; and further imaging results (PDF)
