# Supplementary material for: Deuterated Nanopolymers for Renal and Lymphatic Imaging via Quantitative Deuterium MRI
Source: Nano Lett. 2025 Jan 22;25(5):1758–64. doi: 10.1021/acs.nanolett.4c03036 (PMC11803745; doi:10.1021/acs.nanolett.4c03036)
Supplement: Supplementary file 1 — nl4c03036_si_001.pdf [file nl4c03036_si_001.pdf]

Supporting Information for

Deuterated Nano-Polymers for Renal and Lymphatic Imaging via Quantitative Deuterium MRI

Lisa M. Fries[1,2], *Elton T. Montrazi*[3], *Hyla Allouche-Arnon*[4], *Felipe Opazo*[2,5], *Amnon Bar-Shir*[4], *Lucio Frydman*[3*], *Stefan Glöggler*[1,2*]

[1] NMR Signal Enhancement Group, Max Planck Institute for Multidisciplinary Sciences, Göttingen, Germany

[2]Center for Biostructural Imaging of Neurodegeneration, University Medical Center, Göttingen, Germany

[3]Department of Chemical and Biological Physics, Weizmann Institute of Science, Rehovot, Israel

[4]Department of Molecular Chemistry and Materials Science, Weizmann Institute of Science, Rehovot, Israel

[5]Institute for Neuro- and Sensory Physiology, University Medical Center, Göttingen, Germany

***Corresponding authors:**

Stefan Glöggler, stefan.gloeggler@mpinat.mpg.de

Lucio Frydman, lucio.frydman@weizmann.ac.il

Supporting Methods

Synthesis of PAMAM-G5-Ac-d$_3$

All chemicals were purchased from Sigma Aldrich (St. Louis, MO). The reported synthesis for surface acetylation of PAMAM dendrimers by Kolhatkar et al. was followed. PAMAM-G5 dendrimer (0.216 g, 7.5 µmol) was dissolved in dry methanol under nitrogen atmosphere followed by addition of acetic anhydride (0.125 g, 1.2 µmol) and trimethylamine (0.242 g, 2.4 µmol). The solution was stirred for 14 h at room temperature, after which methanol was evaporated to obtain the crude acetylated PAMAM dendrimers. The crude product was redissolved in water and purified by extensive dialysis against distilled was using dialysis membranes of 2000 MWCO, diameter of 29 mm (Spectrum Laboratories Inc., Rancho Dominguez, CA).

The degree of surface acetylation was quantified using ^{2}H NMR spectroscopy at 14.1 T, as illustrated in Figure S1. Given the natural ^{2}H abundance in H_2O (c_w) of approximately 16.6 mM, the number of deuterium atoms per nano-polymer $N(\,^2\mathrm{H}, \mathrm{PAMAM})$ was calculated.

$$N(\,^2\mathrm{H}, \mathrm{PAMAM}) = \frac{\frac{I_\mathrm{P}}{I_\mathrm{w}} \cdot c_\mathrm{w}}{c_\mathrm{target}} \cdot N_\mathrm{SG} \cdot N_\mathrm{acetyl} = \frac{\frac{2.56}{1} \cdot 16.6\ \mathrm{mM}}{50\ \mathrm{mM}} \cdot 128 \cdot 3 = 326$$

With I_P and I_w being the respective integrals of the PAMAM and HDO signals and c_{target} the targeted ^{2}H concentration within the solution. N_{SG} is the numbers of surface groups of a G5-PAMAM dendrimer and N_{acetyl} the number of ^{2}H atoms per acetyl group.

Synthesis of PAMAM-G5-Ac-d$_3$-atto643

PAMAM-G5-Ac-d$_3$ (36 mg, 1.1 µmol) was dissolved in H_2O (3 ml) and atto-643-NHS-ester (1.0 mg) (Atto-Tech, Siegen, Germany), dissolved in DMF (3 ml), was added to the solution. The mixture was stirred for 24 h at room temperature and then dialyzed against distilled for 4 days, using dialysis membranes of 2000 MWCO, diameter of 29 mm (Spectrum Laboratories Inc., Rancho Dominguez, CA).

High-resolution Nuclear Magnetic Resonance

The synthesized dendrimers were studied with high-resolution ^{1}H and ^{2}H-NMR. NMR experiments were performed on a 14.1 T NMR spectrometers (Bruker, Germany), with the sample temperature stabilized at 298 K. ^{1}H-NMR spectra (600 MHz for 14.1 T) were acquired for all samples prior to the ^{2}H-NMR (92.1 MHz for 14.1 T) spectra, followed by longitudinal (T_1) and transverse (T_2) relaxation-time evaluations. T_1 and T_2 relaxation times were calculated using inversion recovery (IR) and Carr-Purcell-Meiboom-Gill (CPMG) experiments, respectively.

Magnetic Resonance Imaging

Optimization of CSI-SSFP with phantom experiments. All ^{2}H/^{1}H measurements were performed on a horizontal 15.2 T Bruker Biospec scanner running Paravision 6, using 20 mm diameter surface coils tuned to 649.93 (^{1}H) and 99.77 MHz (^{2}H). The CSI-SSFP sequence was optimized for ^{2}H MRI of HDO and PAMAM. For this the T_R was chosen to avoid stop bands for HDO and PAMAM (Fig. S4a) and the flip angle was set to 90° resulting in highest SNR for PAMAM, as T_1~T_2 (Fig. S4b). The offset was adjusted to match that of PAMAM-G5-Ac-d$_3$ to maximize the SNR for the nano-polymer. The specific parameters for the phantom measurements are: 1.9 ppm carrier frequency, T_R = 7.16 ms, flip angle = 90°, in-plane FOV = 42 × 42 mm^2, 26-point, gradient-free FID sampled at 3.2 kHz (out of which the 4 initial points had to be discarded as they were corrupted by the digital filtering), hamming weighted acquisition, averages = 64, repetitions = 2, 32 × 32 encoding matrix, resolution = 1.3 mm.

In vivo renal MRI. Mice were initially anesthetized with 3% isoflurane at 20% O_2 and 80 % N_2, and the levels of isoflurane subsequently lowered to ≈1-1.5% throughout the scans. The surface coil was placed

at the renal region and in each study ^{1}H anatomical images of the mouse's abdomen were taken prior to injection of the deuterated dendrimer. ^{1}H TurboRARE: 10 slices, 0.8 mm thickness, FOVs 42 × 42 mm^2, 512 × 512 encoding matrix. For the kinetic studies one ^{2}H image was taken prior to injection and after injection of 300 nmol PAMAM-G5-Ac-d_3 dissolved in 200 µL PBS ^{2}H images were collected in an interleaved fashion 1 min apart, over the course of 15 min. ^{2}H CSI-SSFP for renal kinetic studies: T_R = 7.16 ms, flip angle = 90°, time = 1 min, in-plane FOV = 42 × 42 mm^2, 16 × 16 encoding matrix, resolution = 2.63 mm, Hamming weighted mode acquisition, averages = 64, repetitions = 2, 20 mm slices accommodating most of the abdomen excited using a 0.63 ms long pulse, with a 26-point, gradient-free FID sampled at 3.2 kHz (out of which the 4 initial points had to be discarded as they were corrupted by the digital filtering). 30 min, 6 h and 24 h after injection high-resolution ^{2}H images of the kidney region were taken. ^{2}H CSI-SSFP for high resolution renal images: T_R = 7.16 ms, flip angle = 90°, time = 24 min, in-plane FOV = 42 × 42 mm^2, 64 × 64 encoding matrix, resolution = 0.656 mm, Hamming weighted acquisition, averages = 64, repetitions = 3, 20 mm slices accommodating most of the abdomen excited using a 0.63 ms long pulse, with a 26-point, gradient-free FID sampled at 3.2 kHz (out of which the 4 initial points had to be discarded as they were corrupted by the digital filtering).

In vivo MRI of Inflamed mice. Mice were initially anesthetized with 3% isoflurane at 20% O_2 and 80 % N_2, and the levels of isoflurane subsequently lowered to ≈1-1.5% throughout the scans. The deuterated dendrimers were injected 10 min prior the first MRI measurement. The surface coil was placed over both legs and in each study, ^{1}H anatomical images were taken. ^{1}H TurboRARE: 10 slices, 1 mm thickness, FOVs 42 × 42 mm^2, 512 × 512 encoding matrix. For the high resolution deuterium images the same parameters were used as for the high-resolution renal studies.

Quantification of PAMAM-G5-Ac-d_3 *in vivo*

To quantify PAMAM-G5-Ac-d_3 in vivo, the internal HDO reference was measured in the bladder, where water content is assumed to be 100%, yielding a natural abundance concentration of HDO at 16.6 mM. In contrast, the HDO signal from the kidney region must be adjusted based on the kidney's water content. Consequently, referencing HDO intensity within the bladder region is deemed more appropriate. To image the bladder, the animal was moved so the bladder was placed almost in the center of the FOV. The bladder was imaged for 2 mice and the intensity of the HDO image was comparable. This involved calculating the average voxel intensity of HDO in the bladder and referencing it to the PAMAM signal intensity in order to calculate the ^{2}H concentration within the region of interest (ROI):

$$c(\,^2\mathrm{H}) = \frac{S_\mathrm{P}^\mathrm{meas}}{S_\mathrm{w}^\mathrm{meas}} \cdot 16.6\ \mathrm{mM} \cdot \left[\frac{S^\mathrm{theo}\left(T_{1,\mathrm{w}}, T_{2,\mathrm{w},}\theta, T_\mathrm{R}\right)}{S^\mathrm{theo}\left(T_{1,\mathrm{P}}, T_{2,\mathrm{P},}\theta, T_\mathrm{R}\right)}\right]$$

Where $S_\mathrm{P}^\mathrm{meas}$ represents the measured signal intensity from PAMAM-G5-Ac-d3 and $S_\mathrm{w}^\mathrm{meas}$ denotes the measured signal intensity from water. S^theo are the theoretical factors that must be considered when referencing this CSI-SSFP signal, dependent on T_1 and T_2 relaxation times, the flip angle θ, and the repetition time T_R.[1] The resulting ^{2}H concentration allows for the determination of the amount of PAMAM-G5-Ac-d_3 within the Region of Interest (ROI).

$$n(\mathrm{PAMAM}) = \frac{c(\,^2\mathrm{H}) \cdot V_\mathrm{voxel} \cdot N_\mathrm{voxel,ROI}}{N(\,^2\mathrm{H}, \mathrm{PAMAM})}$$

Where V_voxel is the volume of the voxel, $N_\mathrm{voxel,ROI}$ is the number of voxel within the ROI.

In vivo Procedures

All animal procedures were approved by the Institutional Animal Care and Use Committee of the Weizmann Institute of Science, which is fully accredited by the AAALAC, the US NIH Office of Laboratory Animal Welfare, and the Israel Ministry of Health. All methods and procedures were performed in accordance to relevant guidelines and regulations. This study is reported in accordance with ARRIVE (Animal Research: Reporting of In Vivo Experiments) guidelines.

Renal imaging. Eight-week-old female SJL/J mice (n=3) were used for in vivo renal MRI measurements.

Local Inflammation Induction. Eight-week-old female SJL/J mice (n=3) were immunized by the subcutaneous injection at the foot pad, of 50 μL of an immunogenic emulsion (in PBS) composed of complete Freund's adjuvant containing 150 μg of Mycobacterium tuberculosis H37Ra. Mice were then placed in their cage for 7 days to develop inflammation.

Data Processing

The CSI-SSFP experiments were reconstructed by zero-filling to 64 x 64 points (or 32 x 32), 2D Fourier transform, and IDEAL fitting as described by Montrazi et al.[2] Data processing utilized MATLAB R2021a; visit https://www.weizmann.ac.il/chembiophys/Frydman_group/software for further processing details. Fat suppression involved filtering the pre-injection ^{2}H image with a 2-D, 3x3 box filter, followed by subtraction from post-injection images as described in the SI of [3].

DLS, Zeta Potential and TEM Measurements

Dynamic Light Scattering (DLS). The particle size and zeta-potential of PAMAM-G5-Ac-d_3 were determined by using a Malvern Zetasizer Nano S system operating at 633 nm at scattering angle of 173°. For Dynamic light scattering (DLS) measurement, the nano-polymer was dissolved in deionized water to make solution with a final concentration of 0.05 mg/mL. The solution was vortexed for 1 min and then sonicated for 3 min. The solution was then filtered through 0.2 μm syringe filters directly into the cell. Three independent measurements were performed.

Zeta Potential. For zeta potential measurement, a solution with a concentration of 0.2 mg/mL was used. The solution was prepared in the same way as the DLS solution and three individual measurements were performed.

Transmission Electron Microscopy (TEM). The TEM images were obtained at 120 kV at a magnification of 57000 x. For size determination the image was analyze with Image J. The sample was diluted to 0.2 mg/ml and sonicated for 2-3 min. Afterwards it was stained with UAc and washed with ddH2O. The grid was glow discharged to make them hydrophil. The respective measurements are shown in Figure S1.

Lymph Node Cells Suspension and Flow Cytometric Analysis

The presence of activated immune cells in immunized lymph node was evaluated by fluorescence-activated cell sorting (FACS). Ten days post immunization, mice were sacrificed and popliteal lymph nodes cells were immediately harvested and suspended in Phosphate buffer solution (PBS) for FACS analysis. Suspended cells were stained with flurochrome labeled monoclonal antibodies for surface antigen detection. PE Cy5 conjugated anti mouse CD8, PE Cy7 conjugated anti mouse CD4, FITC conjugated anti mouse CD19, FITC conjugated anti CD11b, and APC conjugated anti mouse CD45 were purchased from BioLegend and used according to the manufacturer's protocols. All FACS studies were acquired using LSRII (BD Biosciences) cell analyzer flow cytometers running FACSDiva software, and all data were processed by using FlowJo analytical software (Tree Star).

Cell Culture

J774A.1 cells (macrophages isolated from mice with reticulum cell sarcoma) were purchased from Cyticon (Eppelheim, Germany). They were grown in 75 cm^2 flasks using Dulbecco's Modified Eagle Medium (DMEM) high glucose medium, supplemented with fetal bovine serum (10%) and penicillin/streptomycin (1%), at 37 °C in a humidified atmosphere of 5% CO_2. The media was changed each two days, until confluence.

Cellular Staining with Fluorescent-dendrimer

After 80% confluence, J774A.1 cells were detached by scrapping, counted, and incubated at 37°C in a humidified atmosphere of 5% CO_2 in DMEM high glucose medium without FBS for 1 h. Afterwards, the cells were incubated with 1 μg/ml of PAMAM-G5-Ac-d_3-Atto643 and 100 μg/ml Dextran-Alexa594 for 1 h inside the incubator. After treatment, cells were pelleted by centrifugation 1500 x g and washed

3 times with ice-cold 1x Dulbeccos's Phosphate Buffer Saline (DPBS) to remove excess of labelled nano-polymer and Dextran. Then, the cells were chemically fixed with cold 4% paraformaldehyde in phosphate buffer saline (PBS) for 30 minutes at room temperature and finally quenched with 100 mM glycine in PBS. Finally, 1 x 10^6 cells were resuspended in 100 mM glycine in PBS and seeded on poly-L-lysin-coated glass coverslips, and mounted in Mowiol (12 ml of 0.2 M Tris buffer, 6 ml distilled water, 6.0 g glycerol, 2.4 g Mowiol4–88, Merck Millipore) for microscopy.

Microscopy

Endocytosis of PAMAM-G5-Ac-d_3-Atto643 and Dextran-Alexa594 was observed using a STED Expert line microscope (Abberior Instruments GmbH, Göttingen, Germany) on a IX83 inverted microscope (Olympus) equipped with UPLSAPO 100×1.4 NA oil immersion objective (Olympus). For this 561 nm and 640 nm laser were used to image PAMAM-G5-Ac-d_3-Atto643 and Dextran-Alexa594 respectively. Images were analyzed and processed using ImageJ2 v2.14 software.

Supporting Figures

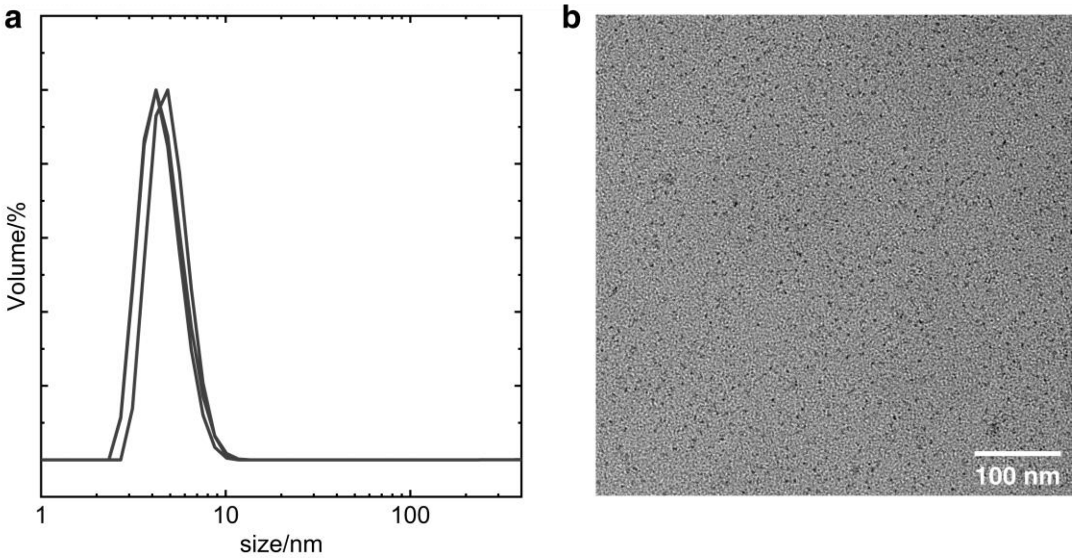

Figure S1. Size determination of PAMAM-G5-Ac-d_3. a DLS measurement with volume distribution in percent. The average size was determined to 4.7 ± 0.2 nm **b** The sample was diluted to 0.2 mg/ml and sonicated for 2-3 min. Afterwards it was stained with UAc and washed with ddH_2O. The grid was glow discharged to make them hydrophil. The average size was determined to 4.6 ± 0.6 nm.

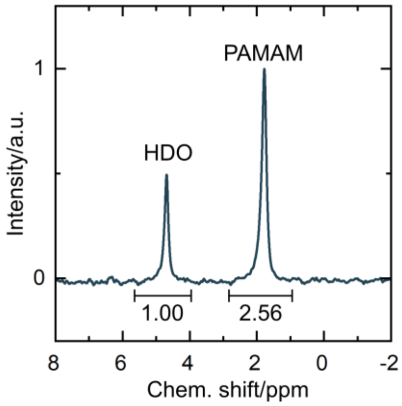

Figure S2. Surface Acetylation of PAMAM-G5-Ac-d_3. ^{2}H NMR measurement at 14.1 T with 360 nM of PAMAM-G5-Ac-d_3 in H_2O.

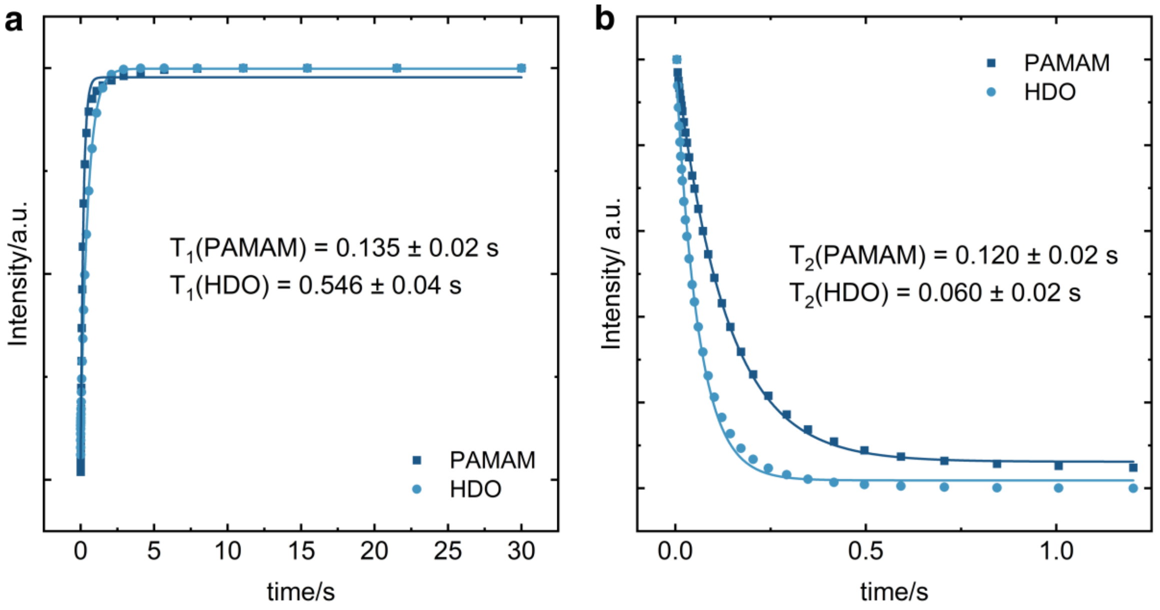

Figure S3. Relaxation time measurements of PAMAM-G5-Ac-d$_3$ and HDO at 600 MHz (14.1 T). a T_1 relaxation time measured via inversion recovery sequence and **b** T_2 relaxation time measured via CPMG.

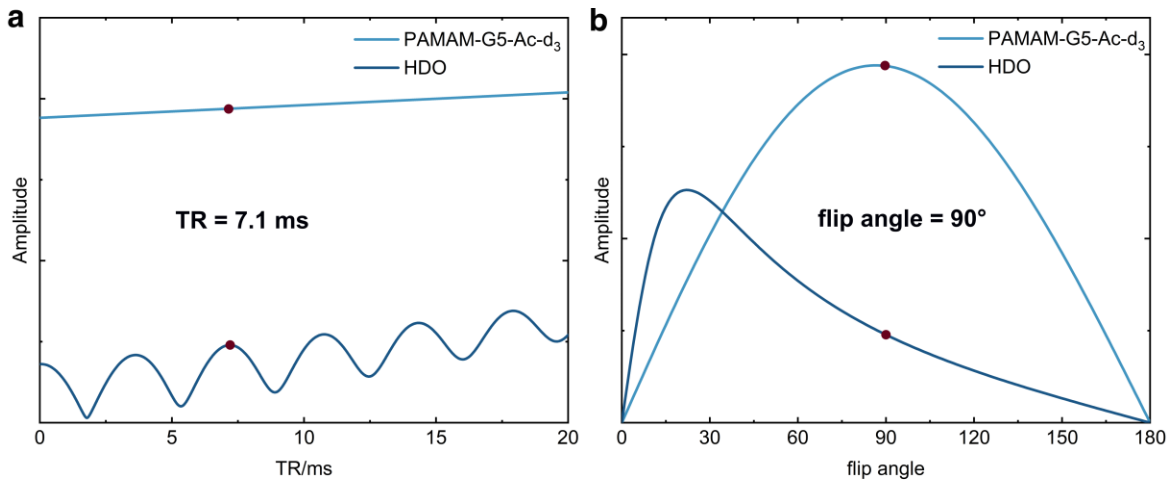

Figure S4. Optimization of SSFP CSI parameters for PAMAM-G5-Ac-d$_3$. a CSI SSFP signal vs T_R for PAMAM-G5-Ac-d$_3$ and HDO. **b** CSI SSFP signal vs flip angle for PAMAM-G5-Ac-d$_3$ and HDO.

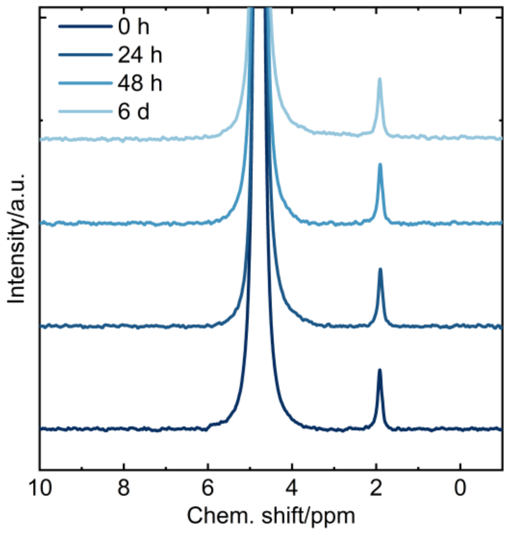

Figure S5. Stability of PAMAM-G5-Ac-d_3 in cell medium. The stability of PAMAM-G5-Ac-d_3 in cell medium was checked using ^2H NMR to exclude unwanted influences of PAMAM-G5-Ac-d_3 on the cell medium and vice versa. As cell medium (Dulbecco's Modified Eagle Medium 61c with 10% fetal bovine serum and 1% Penicillin-Streptomycin with 5% D_2O for locking) was used. The integral of the PAMAM-G5-Ac-d_3 peak is not changing over time.

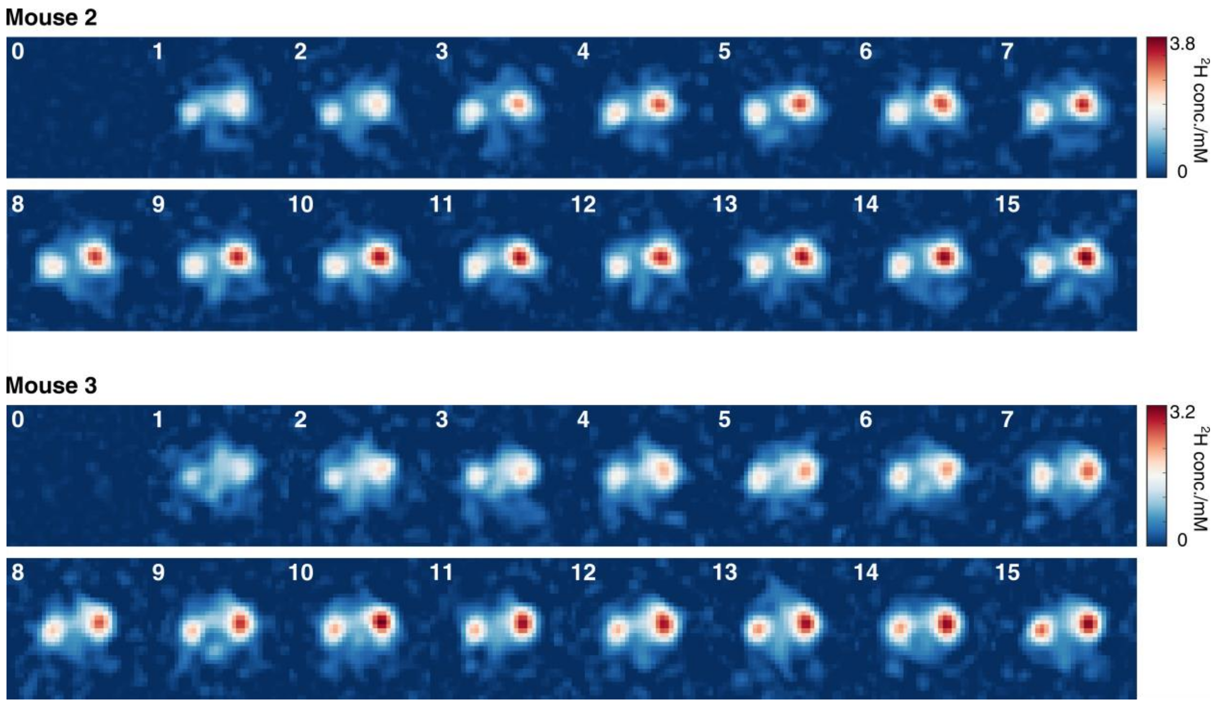

Figure S6. Renal uptake of PAMAM-G5-Ac-d_3 in vivo in mice. ^2H images of PAMAM-G5-Ac-d_3 for real time imaging of renal uptake after intravenous injection of 300 nmol. CSI-SSFP images (16x16 matrix) were acquired every minute with a resolution of 2.6 mm and a FOV of 42x42 mm^2.

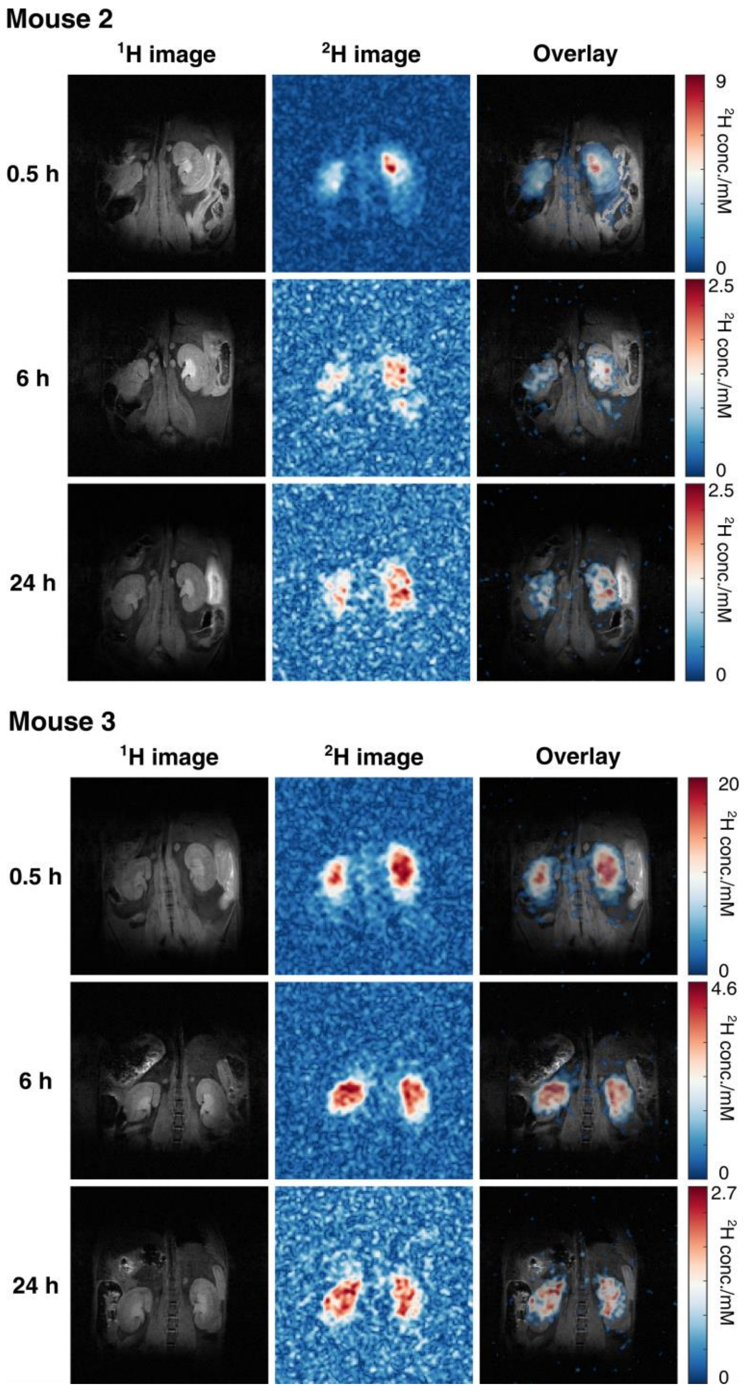

Figure S7. High-resolution deuterium MRI of the kidneys in vivo in mice. ^{1}H anatomical RARE respective ^{2}H images of PAMAM-G5-Ac-d_3, and the $^{1}H/^{2}H$ overlay 0.5 h, 6 h and 24 h after intravenous injection of the dendrimer. All images were referenced to their maximum intensity. CSI-SSFP images (64x64 matrix) were acquired with a resolution of 0.65 mm and a FOV of 42x42 mm^2.

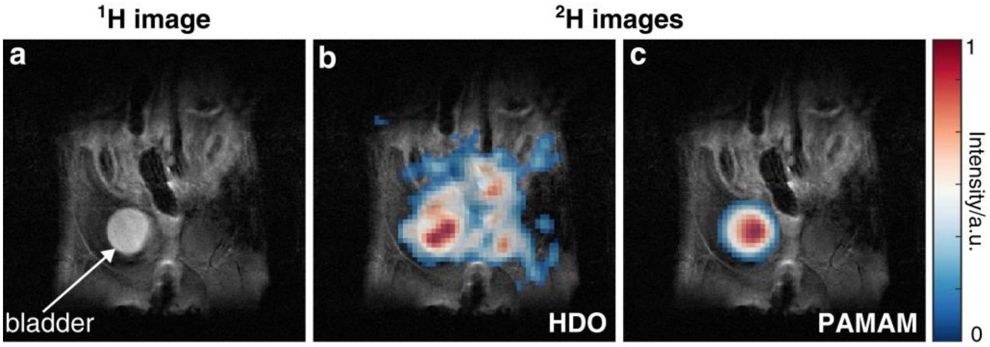

Figure S8. High-resolution deuterium MRI of the bladder in vivo in mice. (a) ^{1}H anatomical RARE image of the representative mouse. (b) ^{2}H image of HDO and (c) PAMAM-G5-Ac-d_3 2h after injection. All images were referenced to their maximum intensity. CSI-SSFP images (64x64 matrix) were acquired with a resolution of 0.65 mm and a FOV of 42x42 mm^2.

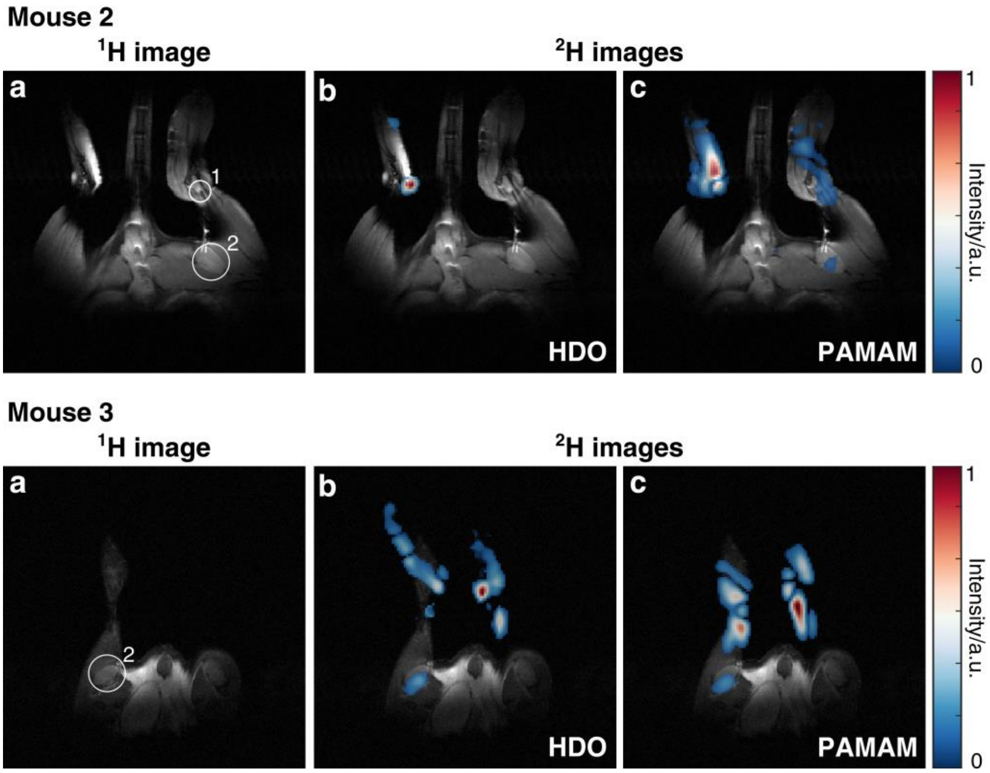

Figure S9. In vivo ^{2}H MRI study of inflamed mice. Seven days post-immunization, each leg of the mice (n=3) was subcutaneously injected with 100 nM PAMAM-G5-Ac-d_3. (a) ^{1}H anatomical image. The lymph nodes (1: popliteal, 2: subiliac) of the inflamed leg are marked with white circles. For mouse 3 the popliteal lymph node was not visible in the anatomical image. (b) $^{1}H/^{2}H$ overlay of the HDO image recorded with CSI-SSFP. (c) $^{1}H/^{2}H$ overlay of the deuterated dendrimer image. ^{2}H images in (b) and (c) were acquired with a resolution of 0.65 mm and a FOV of 42x42 mm^2.

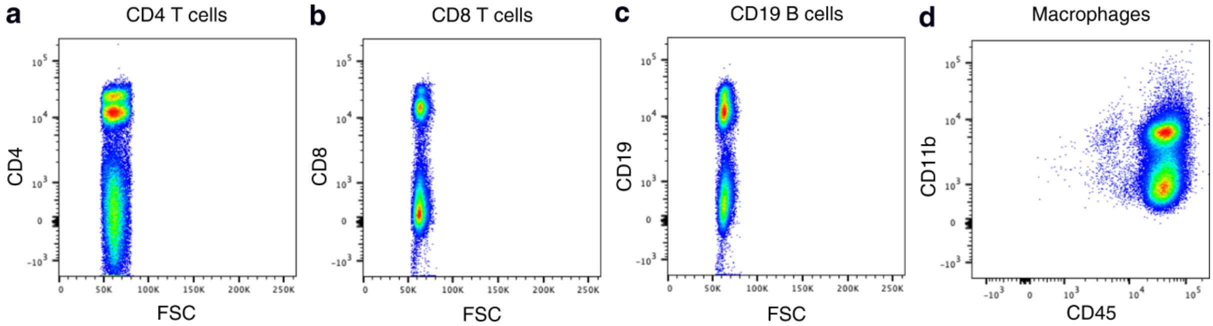

Figure S10. Representative dot blots of FACS analysis of cells excised from lymph nodes 10 days post-immunization. Dot graph representing the lymphatic distribution of activated subtypes of immune cells, including, CD4 T cells (a), CD8 T cells (b), CD19 expressing B cells (c), and CD11b+/ CD45+ macrophages (d).

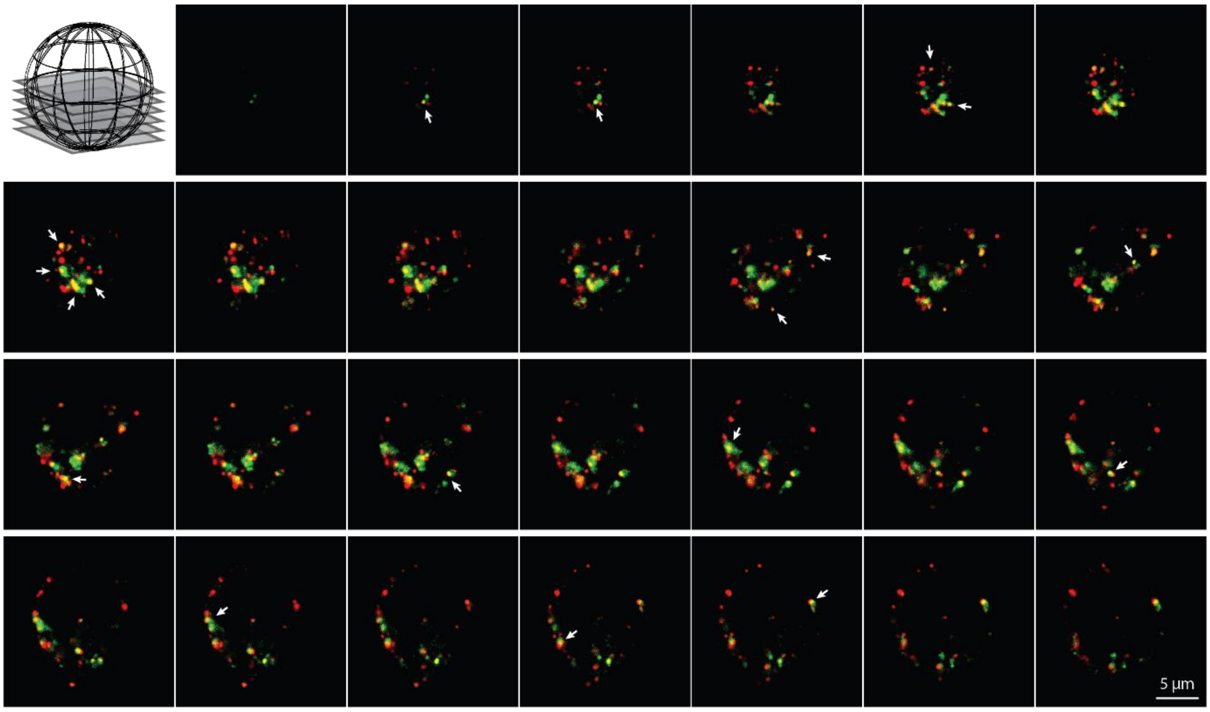

Figure S11. Confocal scanning microscopy on human Macrophage cell line. Live staining was performed on J774A.1 cell line with 1 µg/ml of PAMAM-G5-Ac-d3-Atto643 (red in the images) and 100 µg/ml Dextran-Alexa594 (green in the images). Scheme represents that cell volume was scanned in confocal layers of ~600 nm thickness. The series starts at the bottom of the cells and progresses until the cell equator. White arrows indicate co-localization of the signals, demonstrating that 1 µg/ml of PAMAM-G5-Ac-d3-Atto643 was endocytosed together with Dextran-Alexa594. Scale bar represent 5 µm.

References

1. Peters, D. C.; Markovic, S.; Bao, Q.; Preise, D.; Sasson, K.; Agemy, L.; Scherz, A.; Frydman, L., Improving deuterium metabolic imaging (DMI) signal-to-noise ratio by spectroscopic multi-echo bSSFP: A pancreatic cancer investigation. *Magn Reson Med* **2021,** *86* (5), 2604-2617.
2. Montrazi, E. T.; Sasson, K.; Agemy, L.; Peters, D. C.; Brenner, O.; Scherz, A.; Frydman, L., High-sensitivity deuterium metabolic MRI differentiates acute pancreatitis from pancreatic cancers in murine models. *Sci Rep* **2023,** *13* (1), 19998.
3. Montrazi, E. T.; Sasson, K.; Agemy, L.; Scherz, A.; Frydman, L., Molecular imaging of tumor metabolism: Insight from pyruvate- and glucose-based deuterium MRI studies. *Sci Adv* **2024,** *10* (11), eadm8600.
